# Relationships of Risk Factors for Pre-Eclampsia with Patterns of Occurrence of Isolated Gestational Proteinuria during Normal Term Pregnancy

**DOI:** 10.1371/journal.pone.0022115

**Published:** 2011-07-18

**Authors:** Corrie Macdonald-Wallis, Debbie A. Lawlor, Jon Heron, Abigail Fraser, Scott M. Nelson, Kate Tilling

**Affiliations:** 1 MRC Centre for Causal Analyses in Translational Epidemiology, School of Social and Community Medicine, University of Bristol, Bristol, United Kingdom; 2 School of Social and Community Medicine, University of Bristol, Bristol, United Kingdom; 3 Centre for Population and Health Sciences, University of Glasgow, Glasgow, United Kingdom; Institute of Clinical Effectiveness and Health Policy, Argentina

## Abstract

**Background:**

Isolated gestational proteinuria may be part of the pre-eclampsia disease spectrum. Confirmation of its association with established pre-eclampsia risk factors and higher blood pressure in uncomplicated pregnancies would support this concept.

**Methods:**

Data from 11,651 women from the Avon Longitudinal Study of Parents and Children who had a term live birth but did not have pre-existing hypertension or diabetes or develop gestational diabetes or preeclampsia were used. Proteinuria was assessed repeatedly (median 12 measurements per woman) by dipstick and latent class analysis was used to identify subgroups of the population with different patterns of proteinuria in pregnancy.

**Results:**

Higher maternal pre-pregnancy body mass index (BMI), younger age, nulliparity and twin pregnancy were independently associated with increased odds of any proteinuria in pregnancy. Women who experienced proteinuria showed five patterns: proteinuria in early pregnancy only (≤20 weeks gestation), and onset at 21–28 weeks, 29–32 weeks, 33–36 weeks and ≥37 weeks gestation. There were higher odds of proteinuria onset after 33 weeks in obese women and after 37 weeks in nulliparous women compared with normal weight and multiparous women respectively. Smoking in pregnancy was weakly negatively associated with odds of proteinuria onset after 37 weeks. Twin pregnancies had higher odds of proteinuria onset from 29 weeks. In women with proteinuria onset after 33 weeks blood pressure was higher in early pregnancy and at the end of pregnancy.

**Conclusions:**

Established pre-eclampsia risk factors were related to proteinuria occurrence in late gestation in healthy term pregnancies, supporting the hypothesis that isolated gestational proteinuria may represent an early manifestation of pre-eclampsia.

## Introduction

Pre-eclampsia is a systemic syndrome affecting cardiovascular, renal and hepatic systems and is associated with increased maternal and perinatal morbidity and mortality [1]–[4]. Proteinuria is the main finding used to distinguish pre-eclampsia from the lower risk conditions of gestational and chronic hypertension in pregnancy, although other symptoms may also indicate the presence of the disease in the absence of proteinuria. The current International Society for the Study of Hypertension in Pregnancy (ISSHP) research definition of pre-eclampsia is systolic blood pressure ≥140mmHg or diastolic blood pressure ≥90mmHg with proteinuria of at least 1+ on urine dipstick occurring on 2 occasions after 20 weeks gestation, whereas that for gestational hypertension is the same criteria for high blood pressure but without co-occurrence of proteinuria [5].

Whether isolated gestational proteinuria (i.e. without concomitant high blood pressure) is part of the pre-eclampsia disease continuum is unclear. The classic biological markers of pre-eclampsia; soluble fms-like tyrosine kinase 1 (sFlt-1) and soluble endoglin show intermediate increases (between normal and pre-eclampsia) in women with isolated gestational proteinuria [6], [7]. The clinical literature assessing disease progression from isolated proteinuria to pre-eclampsia is limited, however. A case study of 37 women reported progression from isolated gestational proteinuria to full pre-eclampsia in 19 (51%) women [8] and in two retrospective clinical cohort studies of women with eclampsia, 9.8 and 7.5% respectively had proteinuria alone in the week prior to the first convulsion [9], [10].

If isolated gestational proteinuria is indeed a form of mild pre-eclampsia or an early manifestation of it that occurs before blood pressure risk in some women, we would anticipate that established pre-eclampsia risk factors; maternal pre-pregnancy body mass index (BMI), age, nulliparity and multiple pregnancy would all be positively associated with the occurrence of isolated proteinuria [11], and conversely smoking would be protective [12], [13]. Secondly, if disease progression was likely, we would hypothesise that women who experienced isolated gestational proteinuria would have higher blood pressure at the end of pregnancy than women without proteinuria. Lastly, we would anticipate blood pressure in very early pregnancy also to be higher in these women, in keeping with an established enhanced vascular risk that is uncovered by the physiological stress of pregnancy [14]. In the current study we have tested these three hypotheses in a large prospective cohort study which routinely recorded antenatal dipstick proteinuria assessment.

## Methods

The Avon Longitudinal Study of Parents and Children (ALSPAC) is a prospective birth cohort study investigating influences on the health and development of children. The study has been described in full elsewhere^19^ and on the website www.bristol.ac.uk/alspac. Women with expected delivery dates between 1^st^ April 1991 and 31^st^ December 1992 living in Avon during their pregnancy were eligible for recruitment. Information about the women and their pregnancies was obtained by questionnaire and linkage to obstetric medical records. Ethical approval for the study was obtained from the ALSPAC Law and Ethics Committee and from the National Health Service (NHS) Local Ethics Committee. Written consent was obtained from all participants. In total, 14,541 women were enrolled, 13,863 had singleton or twin pregnancies resulting in all live births and 13,644 of these women had data abstracted from obstetric records. We excluded mothers with triplets (N = 3) and quads (N = 1) due to the small numbers and potential that their identity would be known. We further excluded 446 (3.3%) women who had a previous diagnosis of hypertension, 297 (2.2%) women who developed pre-eclampsia in the index pregnancy (derived from repeat measurements of blood pressure and proteinuria throughout pregnancy using the ISSHP definition [5]), 45 (0.3%) women with existing diabetes and 53 (0.4%) women with gestational diabetes; pregnancies unaffected by these conditions will be referred to as normal for the purposes of the manuscript. We restricted analyses to term pregnancies (≥37 weeks gestation), leaving 11,651 women with at least one proteinuria measurement who are included in this study.

All urine dipstick measurements for proteinuria and blood pressure measurements taken routinely as part of antenatal care by midwives, obstetricians or other relevant health professionals, such as general practitioners, were abstracted from obstetric medical records by six trained research midwives. There was no between-midwife variation in mean values of the data abstracted and error rates were consistently <1% in repeated data entry checks. Proteinuria was recorded as either nil, trace, 1+ (30mg/dl), 2+ (100mg/dl) or 3+ or more (>300mg/dl) and there was a median of 12 and interquartile range of 10 to 14 measurements per woman. The gestational age at each visit was derived from the date of measurement and the expected delivery date.

Maternal age at delivery, number of foetuses and offspring sex were abstracted from obstetric records. Questionnaires administered to the women during early pregnancy asked about pre-pregnancy weight and height, parity, highest educational qualification and smoking status. Pre-pregnancy BMI was derived as weight(kg)/height(m)^2^ and categorised according to WHO definitions of underweight (<18.5kg/m^2^), normal (18.5–24.9kg/m^2^), overweight (25.0–29.9kg/m^2^) and obese (≥30.0kg/m^2^). Smoking was classed as “never” for women who did not smoke immediately prior to or during pregnancy; “pre-pregnancy/1^st^ trimester only” for women who smoked immediately prior to pregnancy or in the first 3 months only and “throughout” for women who continued to smoke after the 1^st^ trimester.

### Statistical Analysis

Multiple logistic regression models were used to assess associations of the risk factors of pre-pregnancy BMI, age, parity, smoking, education and number of foetuses with odds of having proteinuria of 1+ or more (compared with nil or trace) at any time during pregnancy. For this analysis we imputed missing covariate data using multivariate multiple imputation. Chained equations were used to produce 20 full datasets and model coefficients were averaged over the datasets, using Rubin's rules [15] to produce standard errors. The outcome, covariates and predictors of missingness were included in prediction models to impute data.

Latent class analysis was used to extract subgroups of women, or classes, with similar patterns of proteinuria occurrence during pregnancy. This analysis is described in File S1 on the journal website. A latent class model was derived for the whole cohort of women (N = 11,651), using 6 binary variables to define the woman's maximum degree of proteinuria in each of 6 periods of gestation: ≤20 weeks, 21–24 weeks, 25–28 weeks, 29–32 weeks, 33–36 weeks and ≥37 weeks, with categories of “nil/trace” and “1+ or more” or recorded as missing if the woman had no proteinuria measurements in that period. The best-fitting model had two latent classes. Further latent class analysis of women who ever had proteinuria of 1+ or more in pregnancy (N = 1,122), with maximum degree of proteinuria in each period of pregnancy defined in three categories as “nil/trace”, “1+” or “2+ or more”, resulted in a model with five latent classes.

To assess the associations of the maternal characteristics with class membership, pseudo-class draws [16] (random sampling from the distributions of class membership probabilities) were used to create 200 datasets. Regression coefficients were averaged over these datasets, with standard errors produced using Rubin's rules [15] to incorporate the uncertainty in each woman's class membership. Multivariable multinomial regression was used to assess associations of the characteristics with odds of belonging to each of Classes 1–5 obtained from latent class analysis of women who had any proteinuria in pregnancy compared with the pre-defined subgroup who never had proteinuria.

Since the five classes for women who ever had proteinuria appeared to represent timing of onset, we also defined subgroups by the first occurrence of proteinuria of 1+ or more as “never”, “≤20 weeks”, “21–28 weeks, “29–32 weeks”, “33–36 weeks” and “37+ weeks”, and used multinomial regression to investigate associations of the risk factors with membership of these subgroups, imputing missing covariate data using multivariate multiple imputation as described above.

To obtain blood pressure at 8 weeks we used the predicted values of linear spline random effects models for changes in systolic and diastolic blood pressure with gestational age, with three knots (indicating changes in slope) at 18, 30 and 36 weeks and baseline set at 8 weeks [17]. Further description of these models is given in File S2 on the journal website. Blood pressure at the end of pregnancy was obtained from the last blood pressure measurement, or treated as missing for women with no blood pressure measurements on or after 37 weeks gestation. The difference between the last blood pressure measurement and predicted blood pressure at 8 weeks was used to calculate absolute blood pressure change across pregnancy. We used pseudo-class draws (as above) and linear regression models to investigate mean differences in blood pressure at 8 weeks and at the end of pregnancy and absolute change in blood pressure between each of the latent classes of women who ever had proteinuria in pregnancy compared with the subgroup of women who never had proteinuria. In Model 1 we did not adjust for any other variables, and in Model 2 we adjusted for each of the maternal characteristics (pre-pregnancy BMI, age, parity, smoking, education and pregnancy type).

We also performed three sensitivity analyses, the first including the 205 women who had pre-eclampsia (defined according to ISSHP criteria) but who were delivered at term in our main analysis dataset, the second restricting to nulliparous women (N = 4827) and the third excluding 455 women who reported a previous diagnosis of kidney disease in a questionnaire administered during pregnancy. Latent class models were fitted in Mplus version 5 and all other analyses were completed using Stata version 11.1.

## Results

The characteristics of all women included in the latent class analysis (N = 11,651), the subset with complete data on all maternal characteristics (N = 8,915) and the subset with complete data on all maternal characteristics and blood pressure variables (N = 8,568) are shown in Table 1. Distributions of characteristics in both of the subsets were similar to those in the full dataset.

**Table 1:** Characteristics of all women with proteinuria measurements and the subsets with complete data on all of the maternal covariables and blood pressure.

Maternal Characteristic	Full dataset	Complete maternal covariable data	Complete maternal covariable and BP data
	(Total N = 11,651)	(N = 8,915)	(N = 8,568)
	(%)/mean (SD)	(%)/mean (SD)	(%)/mean (SD)
**Age (%)**	N = 11651		
15–19	4.8	3.4	3.3
20–24	19.3	16.4	16.4
25–29	38.7	39.8	39.8
30–34	27.6	29.9	30.0
35+	9.6	10.4	10.5
**Pre-pregnancy BMI (%)**	N = 9702		
Underweight	5.1	4.9	4.7
Normal	74.8	75.2	75.2
Overweight	14.9	14.8	15.0
Obese	5.2	5.1	5.1
**Parity (%)**	N = 10824		
Nulliparous	44.6	44.7	44.7
Multiparous	55.4	55.3	55.3
**Smoking in pregnancy (%)**	N = 10940		
Never	66.2	69.0	69.0
Pre-pregnancy/1^st^ trimester	13.9	13.2	13.4
Throughout	20.0	17.8	17.6
**Highest qualification (%)**	N = 10489		
CSE/Vocational	29.9	27.5	27.4
O Level	34.5	35.1	35.2
A Level	22.5	23.5	23.7
Degree	13.1	13.9	13.8
**Pregnancy type (%)**	N = 11651		
Male singleton	50.7	50.4	50.4
Female singleton	48.5	48.8	48.8
Twin	0.8	0.8	0.8
**BP at 8 weeks (mmHg)**	N = 11644	N = 8910	
SBP	112.20 (6.48)	112.20 (6.40)	112.21 (6.44)
DBP	66.03 (4.33)	66.02 (4.27)	66.02 (4.29)
**BP at end of pregnancy (mmHg)**	N = 11152	N = 8569	
SBP	119.46 (13.18)	119.39 (13.18)	119.38 (13.18)
DBP	73.80 (10.54)	73.75 (10.49)	73.75 (10.49)
**Absolute change in BP (mmHg)**	N = 11150	N = 8568	
SBP	7.25 (11.78)	7.18 (11.83)	7.18 (11.83)
DBP	7.76 (9.50)	7.73 (9.50)	7.73 (9.50)

Abbreviations: BP blood pressure; DBP diastolic blood pressure; SBP systolic blood pressure; SD standard deviation.

SBP and DBP at 8 weeks are the predicted values of linear spline random effects models for blood pressure change across pregnancy [17]; SBP and DBP at the end of pregnancy were obtained from the last recorded blood pressure measurement; change across pregnancy is the difference between these two measures.

Single episodes of isolated gestational proteinuria were relatively common with 894 women (7.7%) experiencing proteinuria of 1+ or more on one occasion only, 157 (1.3%) on 2 occasions and 71 (0.6%) on more than 2 occasions. With respect to severity of proteinuria 972 women (8.3%) had an occurrence of 1+ proteinuria, 176 (1.5%) experienced 2+ and 38 (0.3%) experienced 3+ or more. Compared to reference categories the odds of experiencing proteinuria were higher if the woman was overweight or obese, aged <20 or 20–24 years, nulliparous or pregnant with twins (Table 2).

**Table 2:** Odds ratios for ever having proteinuria of 1+ or more at any time during pregnancy in mutually-adjusted model (N = 11,651)^a^.

Maternal characteristic	Odds ratio	95% confidence interval
**Pre-pregnancy BMI (kg/m^2^)**		
Underweight	0.88	(0.63, 1.22)
Normal	1	-
Overweight	1.30	(1.09, 1.55)
Obese	1.75	(1.34, 2.28)
**Age (yrs)**		
<20	1.49	(1.13, 1.97)
20–24	1.33	(1.13, 1.58)
25–29	1	-
30–34	1.00	(0.84, 1.17)
35+	0.96	(0.75, 1.22)
**Parity**		
Nulliparous	1	-
Multiparous	0.87	(0.76, 1.00)
**Smoking during pregnancy**		
Never	1	-
Pre-pregnancy/1^st^ trimester	1.01	(0.83, 1.23)
Throughout	1.09	(0.93, 1.28)
**Highest qualification**		
CSE/vocational	1.09	(0.92, 1.28)
O level	1	-
A level	1.03	(0.86, 1.24)
Degree	0.83	(0.65, 1.06)
**Pregnancy type**		
Male singleton	1	-
Female singleton	1.08	(0.96, 1.23)
Twin	3.09	(1.88, 5.08)

a Missing covariate data was imputed using multivariate multiple imputation.

**Figure 1:**
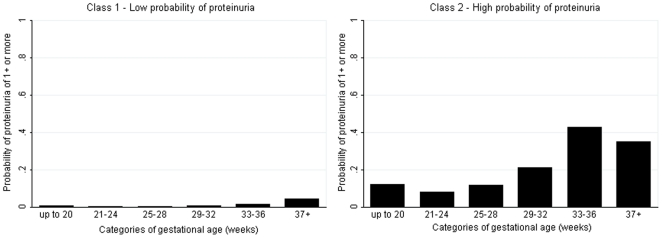
Probability of proteinuria of 1+ or more by gestational age in the latent class model for all women (N = 11,651).

**Figure 2:**
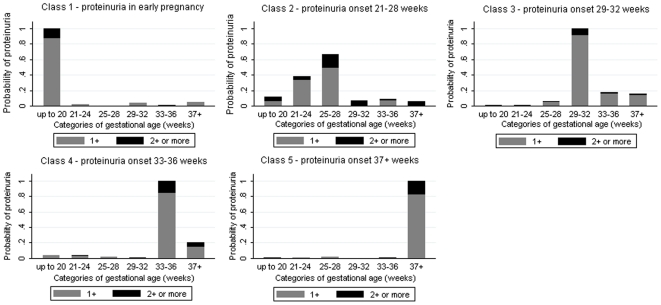
Probability of proteinuria in each category of gestational age in the latent class model for women who experienced 1+ or more episodes of proteinuria in pregnancy (N = 1,122).

Latent class analysis of the full cohort produced two classes of women (Table 3). The first class (N = 11,511, 98.8%) had a low probability of proteinuria throughout gestation (Figure 1). The second class (N = 140, 1.2%) had a higher probability of proteinuria, which was greatest in late pregnancy (Figure 1). Restriction of the analyses to only those women who experienced proteinuria during pregnancy, N = 1,122, produced five latent classes related to gestational age (i.e. timing of occurrence in pregnancy) (Table 3). The probability of experiencing proteinuria in each of these gestational periods is shown in Figure 2, with clear differential patterns regarding the timing of onset of proteinuria identified by the latent class analysis.

**Table 3:** Description of the latent classes in analysis of the whole cohort and of the subset who ever had proteinuria assigning women to their most likely class.

Latent Class	N (%)	N with complete data on all covariates (%)	Description
**Whole cohort analysis:**			
Class 1	11511 (98.8)	8831 (99.1)	Low probability of proteinuria
Class 2	140 (1.2)	84 (0.9)	High probability of proteinuria
**Analysis of women who ever had proteinuria:**			
Class 1	101 (9.0)	77 (9.7)	Proteinuria only in early pregnancy
Class 2	104 (9.3)	71 (8.9)	Onset of proteinuria 21–28 weeks
Class 3	137 (12.2)	95 (11.9)	Onset of proteinuria 29–32 weeks
Class 4	276 (24.6)	173 (21.7)	Onset of proteinuria 33–36 weeks
Class 5	504 (44.9)	380 (47.7)	Onset of proteinuria 37 weeks gestation or later

The associations between the maternal characteristics and belonging to each of the five classes are shown in Table 4, with the probabilities of belonging to each class by maternal characteristic shown in Table S3. Compared with those in the reference categories, women aged <20 or 20–24 years and nulliparous women were more likely to belong to Class 1 (proteinuria ≤20 weeks). There were no strong associations of the risk factors with membership of Class 2 (proteinuria onset 21–28 weeks). Women with twin pregnancies and women who smoked pre-pregnancy/1st trimester were more likely to belong to Class 3 (proteinuria onset 29–32 weeks). Obese women and those with twins were more likely to belong to Class 4 (proteinuria onset between 33 and 36 weeks). Notably there was an increasing trend in odds of belonging to Class 5 (proteinuria onset 37 weeks or later) with higher BMI category and nulliparous women were also more likely to belong to this class. There was also some evidence that women who smoked pre-pregnancy/1^st^ trimester or throughout pregnancy were less likely to belong to Class 5 than those who never smoked. When we defined the subgroups by the onset of proteinuria measurement and analysed by multinomial regression analysis, the results were similar, further supporting the interpretation of these classes as representing the timing of onset of proteinuria (Table S4).

**Table 4:** Odds ratios for class membership (analysis of women who ever had proteinuria) compared with women who never had proteinuria in multivariable multinomial regression model (N = 8,915).

Maternal Characteristic	Class 1^a^ (0.84%)		Class 2^a^ (0.87%)		Class 3^a^ (1.08%)		Class 4^a^ (1.91%)		Class 5^a^ (4.23%)	
	“Onset ≤20 weeks”		“Onset 21–28 weeks”		“Onset 29–32 weeks”		“Onset 33–36 weeks”		“Onset 37+ weeks”	
	Odds ratio	95% CI	Odds ratio	95% CI	Odds ratio	95% CI	Odds ratio	95% CI	Odds ratio	95% CI
**Pre-pregnancy BMI (kg/m^2^)**										
Underweight	1.11	(0.41, 3.01)	0.33	(0.05, 2.32)	0.35	(0.07, 1.67)	1.53	(0.82, 2.85)	0.50	(0.25, 0.98)
Normal	1	-	1	-	1	-	1	-	1	-
Overweight	1.10	(0.56, 2.14)	1.31	(0.71, 2.44)	0.84	(0.46, 1.57)	1.29	(0.85, 1.97)	1.30	(0.99, 1.72)
Obese	1.60	(0.65, 3.97)	1.68	(0.71, 3.99)	1.50	(0.68, 3.31)	2.28	(1.34, 3.88)	1.90	(1.27, 2.82)
***P*** ** for all classes** = 0.006										
**Age (yrs)**										
<20	6.53	(2.58, 16.55)	2.26	(0.79, 6.46)	1.28	(0.41, 4.00)	0.80	(0.29, 2.20)	1.20	(0.68, 2.13)
20–24	2.46	(1.30, 4.66)	1.42	(0.73, 2.75)	1.20	(0.67, 2.14)	1.25	(0.81, 1.93)	1.12	(0.82, 1.52)
25–29	1	-	1	-	1	-	1	-	1	-
30–34	0.79	(0.41, 1.53)	1.11	(0.60, 2.04)	0.91	(0.54, 1.53)	1.09	(0.74, 1.60)	1.06	(0.82, 1.38)
35+	0.75	(0.29, 1.94)	0.59	(0.20, 1.76)	0.58	(0.24, 1.40)	0.96	(0.54, 1.72)	1.24	(0.86, 1.78)
***P*** ** for all classes** = 0.044										
**Parity**										
Nulliparous	1	-	1	-	1	-	1	-	1	-
Multiparous	1.68	(1.00, 2.84)	1.16	(0.69, 1.96)	1.53	(0.97, 2.41)	1.10	(0.79, 1.53)	0.62	(0.49, 0.77)
***P*** ** for all classes** <0.001										
**Smoking during pregnancy**										
Never	1	-	1	-	1	-	1	-	1	-
Pre-pregnancy/1^st^ trimester	0.44	(0.17, 1.12)	1.33	(0.67, 2.63)	1.79	(1.04, 3.07)	1.31	(0.85, 2.01)	0.74	(0.53, 1.03)
Throughout	0.73	(0.38, 1.38)	1.46	(0.80, 2.64)	1.22	(0.71, 2.12)	0.96	(0.63, 1.47)	0.77	(0.57, 1.04)
***P*** ** for all classes** = 0.084										
**Highest qualification**										
CSE/vocational	0.96	(0.53, 1.76)	1.20	(0.69, 2.10)	1.56	(0.92, 2.63)	1.39	(0.95, 2.03)	1.08	(0.82, 1.41)
O level	1	-	1	-	1	-	1	-	1	-
A level	1.38	(0.73, 2.62)	0.63	(0.28, 1.42)	1.63	(0.93, 2.88)	1.16	(0.76, 1.78)	1.05	(0.80, 1.39)
Degree	1.14	(0.48, 2.71)	1.13	(0.50, 2.59)	0.89	(0.37, 2.13)	0.70	(0.38, 1.32)	0.76	(0.52, 1.10)
***P*** ** for all classes** = 0.281										
**Pregnancy type**										
Male singleton	1	-	1	-	1	-	1	-	1	-
Female singleton	1.02	(0.63, 1.63)	1.00	(0.61, 1.62)	1.05	(0.69, 1.60)	1.35	(0.98, 1.86)	0.97	(0.79, 1.20)
Twin	-b	-b	4.64	(0.91, 23.64)	5.24	(1.53, 17.93)	8.64	(3.77, 19.81)	1.39	(0.43, 4.50)
***P*** ** for all classes** <0.001										

Abbreviations: CI confidence interval; BMI body mass index.

a Versus never had proteinuria (N = 8,119, 91.07%).

b Odds ratio could not be estimated as there were too few women with this characteristic in this class.

**Figure 3:**
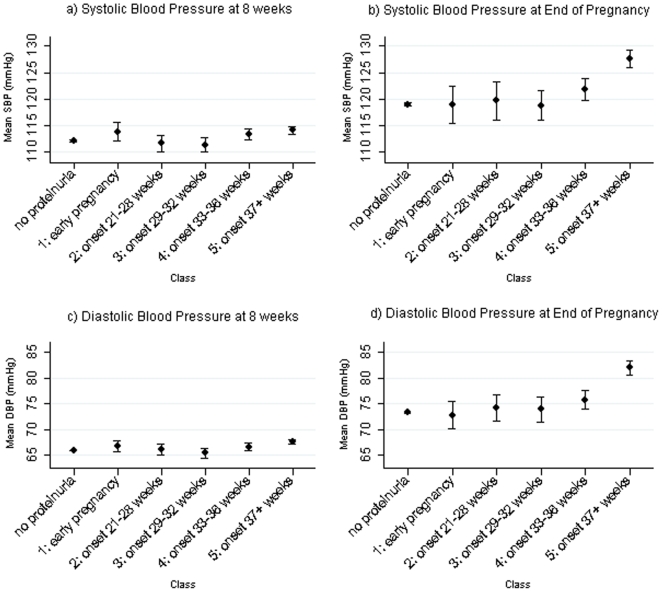
Mean and 95% confidence intervals of early and late pregnancy blood pressure by classes of women who ever had proteinuria and the subgroup without proteinuria. SBP and DBP at 8 weeks are the predicted values from linear spline random effects models for blood pressure change across pregnancy [17]; SBP and DBP at the end of pregnancy were obtained from the last recorded blood pressure measurement provided that this occurred at ≥37 weeks. The 8,568 women with complete data on all maternal characteristics and blood pressure measures were included.

Assessment of the association of these proteinuric classes with blood pressure at the beginning and end of pregnancy is shown in Table 5 and Figure 3. Of the five different classes it was striking that women in Class 5 (proteinuria onset 37 weeks or later) had higher SBP and DBP at 8 weeks and also at the end of pregnancy and overall experienced a greater absolute rise in SBP and DBP across pregnancy compared with women with no proteinuria in fully adjusted models (Table 5).

**Table 5:** Mean differences (95% confidence interval) in blood pressure at the beginning and end of pregnancy and absolute change in blood pressure for each of the classes of women who ever had proteinuria compared with those who did not (N = 8,568).

Class	SBP at 8 weeks	SBP at end of pregnancy	SBP change across pregnancy	DBP at 8 weeks	DBP at end of pregnancy	DBP change across pregnancy
(comparison group is women who never had proteinuria; 91.07%)	Mean difference(95% CI)	Mean difference(95% CI)	Mean difference(95% CI)	Mean difference(95% CI)	Mean difference(95% CI)	Mean difference(95% CI)
**Class 1** (0.84%)						
“Onset ≤20 weeks”						
Model 1	1.76 (0.17, 3.34)	−0.05 (−3.28, 3.19)	−1.80 (−4.74, 1.14)	0.91 (−0.15, 1.97)	−0.56 (−3.14, 2.02)	−1.47 (−3.80, 0.87)
Model 2	1.74 (0.24, 3.25)	0.14 (−3.04, 3.32)	−1.60 (−4.53, 1.33)	1.01 (0.02, 2.01)	−0.30 (−2.83, 2.22)	−1.32 (−3.63, 1.00)
**Class 2** (0.87%)						
“Onset 21–28 weeks”						
Model 1	−0.46 (−2.01, 1.09)	0.73 (−2.45, 3.91)	1.19 (−1.67, 4.05)	0.16 (−0.87, 1.19)	0.86 (−1.67, 3.38)	0.69 (−1.59, 2.98)
Model 2	−0.83 (−2.31, 0.66)	0.31 (−2.82, 3.44)	1.14 (−1.71, 3.99)	0.05 (−0.91, 1.02)	0.68 (−1.78, 3.14)	0.63 (−1.63, 2.89)
**Class 3** (1.08%)						
“Onset 29–32 weeks”						
Model 1	−0.72 (−2.09, 0.64)	−0.17 (−2.98, 2.64)	0.55 (−1.98, 3.09)	−0.43 (−1.35, 0.49)	0.59 (−1.63, 2.82)	1.03 (−0.98, 3.03)
Model 2	−0.74 (−2.04, 0.56)	−0.11 (−2.87, 2.65)	0.63 (−1.90, 3.16)	−0.33 (−1.19, 0.54)	0.80 (−1.37, 2.98)	1.13 (−0.86, 3.11)
**Class 4** (1.91%)						
“Onset 33–36 weeks”						
Model 1	1.29 (0.28, 2.30)	2.84 (0.77, 4.91)	1.55 (−0.30, 3.41)	0.73 (0.06, 1.40)	2.44 (0.80, 4.08)	1.71 (0.23, 3.19)
Model 2	0.95 (−0.01, 1.92)	2.42 (0.39, 4.46)	1.47 (−0.39, 3.33)	0.54 (−0.10,1.17)	2.16 (0.56, 3.76)	1.62 (0.15, 3.09)
**Class 5** (4.23%)						
“Onset 37+ weeks”						
Model 1	2.06 (1.39, 2.73)	8.66 (7.29, 10.03)	6.60 (5.37, 7.84)	1.73 (1.29, 2.18)	8.62 (7.54, 9.70)	6.89 (5.91, 7.87)
Model 2	1.52 (0.88, 2.16)	7.86 (6.52, 9.21)	6.34 (5.11, 7.58)	1.36 (0.94, 1.78)	7.92 (6.86, 8.98)	6.56 (5.59, 7.54)

Abbreviations: SBP systolic blood pressure; DBP diastolic blood pressure; CI confidence interval.

SBP and DBP at 8 weeks are the predicted values of linear spline random effects models for blood pressure change across pregnancy [17]; SBP and DBP at the end of pregnancy were obtained from the last recorded blood pressure measurement; change across pregnancy is the difference between these two measures.

Model 1 is unadjusted; Model 2 is adjusted for maternal pre-pregnancy BMI, age, parity, smoking, education and pregnancy type.

In the sensitivity analysis in which we included women who had pre-eclampsia during the pregnancy, but delivered at term (N = 205 women added), and all other elements of our eligible cohort remained, results were largely the same as those already presented when these women were not included. The main exception was that in the latent class analyses of women who ever had proteinuria an additional sixth class (to the five produced in the main analyses) which included a heterogeneous group of women with proteinuria onset 21–24 weeks, those with proteinuria throughout pregnancy and those with repeated proteinuria of 2+ or more (representing 3.6% of women with at least one episode of proteinuria) was produced. Also instead of the class with proteinuria onset 21–28 weeks there was a class with proteinuria onset 25–28 weeks (6.3%). Associations of the maternal risk factors with membership of classes were comparable with the main analysis, though the reduced odds of proteinuria in later pregnancy in women who smoked in early or throughout pregnancy was strong in this sensitivity analysis including women with pre-eclampsia. In terms of characteristics associated with the new/different classes, maternal age <20 years was associated with greater odds of belonging to the additional sixth class and women with twin pregnancies were more likely to belong to the class with proteinuria onset 25–28 weeks compared with singleton pregnancies. Repeating the analysis with restriction to nulliparous women (N = 5,997 multiparous women excluded) or excluding women with a previous diagnosis of kidney disease (N = 455) produced results consistent with the main analysis.

## Discussion

In a large cohort of women we demonstrate that established risk factors for pre-eclampsia are associated with isolated gestational proteinuria. Furthermore, using a novel latent class method, we establish that these risk factors are related to different patterns of the onset of proteinuria, with this occurring earlier in twin pregnancy than in relation to obesity or nulliparity. Lastly, women who develop proteinuria in late gestation commence pregnancy with higher systolic and diastolic blood pressure and this relative increase is maintained across gestation. Our ability to exclude women with pre-existing hypertension or diabetes or those who subsequently develop gestational diabetes or pre-eclampsia, further enhances their validity as our associations are not driven by such women. Collectively, our results support the hypotheses that isolated gestational proteinuria is part of the disease spectrum of pre-eclampsia, that disease progression is biologically plausible and that women enter pregnancy with an established risk profile which is determined genetically or through lifestyle but subsequently manifests clinically as pregnancy complications including that of isolated gestational proteinuria.

Of the studied maternal risk factors young maternal age, higher pre-pregnancy BMI, nulliparity and twin pregnancy were all associated with increased odds of experiencing proteinuria. Using latent class analysis we identified that the individual maternal risk factors were associated with different patterns of proteinuria onset. Specifically younger women were more likely to experience proteinuria in early pregnancy, but not later in pregnancy, agreeing with evidence that younger maternal age is not related to pre-eclampsia risk when adjusting for parity [18]. Maternal obesity was associated with proteinuria onset from 33 weeks onwards, nulliparity from 37 weeks and twin pregnancy from 29 weeks. These patterns of onset coincide with our previously reported temporal analysis of the rise in blood pressure across gestation [17]. In our previous study of blood pressure change during pregnancy conducted in this cohort we found that in obese women blood pressure began to rise at 30 weeks gestation as compared to 18 weeks for normal weight women, and also rose more rapidly [17]. In nulliparous women, although blood pressure was higher than for multiparous women throughout pregnancy, there were faster gains in diastolic blood pressure from 30 weeks and systolic blood pressure from 36 weeks onwards. In twin pregnancies again the rise in blood pressure began at around 30 weeks and was more rapid than singleton pregnancies [17]. Thus, our two studies together suggest that established risk factors for pre-eclampsia are associated with blood pressure change and isolated gestational proteinuria in normal term pregnancies.

The striking similarity between the timing of the observed increases in blood pressure (in our previous study) and the timing of onset of proteinuria (in our current study) which is apparent in obese women, nulliparous women and women pregnant with twins may reflect two potential non-mutually exclusive mechanisms. Firstly, that the release of factors from the placenta influences both blood pressure increases and glomerular endothelial dysfunction; and/or, secondly, that the rise in blood pressure causes previously negligible proteinuria to progress to clinically significant levels. Indeed in pre-eclampsia, proteinuria is thought to stem from the placental production of high levels of sFlt-1, leading to inactivation of free vascular endothelial growth factor (VEGF) on podocytes and glomerular endotheliosis [19]. Furthermore proteinuria may be made more severe by high blood pressure, due to increased pressure on the glomerular endothelium [19].

In agreement with our findings, BMI has also been previously associated with an increased risk of proteinuria in pregnancy [20], [21] and uncomplicated twin pregnancies exhibit both higher protein∶creatinine ratios at 34–38 weeks and absolute change across gestations compared with singleton pregnancies [22]. Our observed lack of relationship between twin pregnancies and onset of proteinuria after 37 weeks, is potentially attributable to 75% of the twin pregnancies being delivered at 37 or 38 weeks.

Smoking is known to be protective against pre-eclampsia and in accordance with this smoking either pre-pregnancy/first trimester only or throughout pregnancy were both weakly associated with reduced odds of proteinuria onset after 37 weeks. This effect was somewhat stronger when women with pre-eclampsia were included in analyses. Our previous findings showed that women who smoked throughout pregnancy had consistently lower blood pressure in pregnancy, while those who smoked only in the first trimester had similar patterns of blood pressure change in late pregnancy to those who never smoked [17]. Thus, it is plausible that the reduction in risk of proteinuria onset in late pregnancy in smokers occurs via a different mechanism to the decreased blood pressure.

In keeping with an increased risk of disease progression to pre-eclampsia [8], we found that blood pressure was higher at the end of pregnancy in women who had onset of proteinuria after 33 weeks gestation. Furthermore in these same classes of women blood pressure was higher in early pregnancy. This suggests that any increased risk of hypertensive disorders of pregnancy in these women, partly reflects genetic predisposition or pre-pregnancy lifestyle rather than being solely attributable to pregnancy related factors. Consistent with this, outside of pregnancy, obesity, blood pressure and older age are all positively associated with proteinuria [23]–[25].

Less than 10% of women experienced proteinuria during pregnancy, and only 2% on more than one occasion, so we could not model longitudinal changes in proteinuria. Aggregating proteinuria measurements over periods of gestational age enabled us to use latent class analysis to define subgroups of women by patterns in the timing and severity of proteinuria and relate these subgroups to pre-eclampsia risk factors. All models had entropy values ≥0.89, indicating that women had high probabilities of belonging to their most likely class and the classes represented distinct clusters of patterns. Furthermore, we were able to still include women who did not have proteinuria measurements in all periods of gestation by assigning them a probability of class membership reflecting the uncertainty due to the missing data. There is increasing awareness of the benefits of latent class analysis for repeat measurements, with recent characterisation of patterns of clinical symptoms such as wheeze [26] and back pain [27] reported in repeated questionnaires. We have extended the utility of this technique to demonstrate that it can also be valuable in analysis of repeated routine clinical measurements on a categorical scale, measured at different time points for each individual, particularly where some values are uncommon.

Our study has a number of strengths including its large size, detailed repeat measurements and our novel application of latent class analysis, to overcome the methodological challenges that arise due to proteinuria being a sparse discrete categorical variable. In addition to these significant strengths we do, however, acknowledge that our study was limited by the use of routine qualitative dipstick measurements of proteinuria, which are less accurate than 24 hour collections or protein/creatinine ratio and more influenced by urine concentration. However, until recently dipstick was the only method of feasibly assessing proteinuria in routine antenatal practice and is currently being used to screen for pre-eclampsia in the UK and most other countries, and thus our results are applicable to clinical practice. In clinical practice dipstick measurements indicating pre-eclampsia should be confirmed by a timed urine collection (e.g. 24 hour urine) or protein/creatinine ratio [5]. We do not have such measurements available in our study and it would be valuable to examine whether our findings replicate in studies that do have the ability to confirm proteinuria and that have quantitative measurements. Since the blood pressure and proteinuria measurements were routinely collected at antenatal visits the methods of measurement could not be standardised between the clinics included in the study. However, this also means that our data are reflective of a clinical setting, where women are assessed by different staff in different locations (e.g. home visits, primary care, secondary care) throughout their pregnancy. Also, we recognise that there are other aetiologies of isolated gestational proteinuria which are unrelated to pre-eclampsia and were unable to exclude these from our data, but in clinical practice other causes should be considered in order to rule these out. We also assumed that women without any occurrences of proteinuria on urine dipstick tests would not have had proteinuria at times in pregnancy when they were not tested, but this seems plausible given the high number of repeated measurements per woman.

### Conclusions

In this study we demonstrate that isolated gestational proteinuria is relatively common affecting ∼10% of women, and that it exhibits positive associations with the known maternal pre-eclampsia risk factors; obesity, nulliparity, multiple pregnancy and blood pressure. Our observation that isolated gestational proteinuria and pre-eclampsia share the same maternal antecedents supports the concept that they are part of the same disease spectrum. However, these findings require confirmation by studies with quantitative accurate measurements of proteinuria. While our findings are not sufficient to support the changing of clinical management of women who experience isolated gestational proteinuria, they indicate a clear need for future studies which assess the potential for progression to pre-eclampsia in women with isolated gestational proteinuria and to evaluate the health benefit of more intense antenatal monitoring in those women with isolated gestational proteinuria.

## Supporting Information

File S1 **Supplemental Methods: Description of the latent class analysis.**
(DOC)

File S2 **Supplemental Methods: Development of the linear spline random effects models for blood pressure.**
(DOC)

Table S3 **Probabilities of class membership (analysis of women who ever had proteinuria) and membership of the subgroup of women who never had proteinuria in extreme categories of the maternal risk factors from the multivariable multinomial regression model (N = 8915).**
(DOC)

Table S4 **Odds ratios for membership of subgroups defined by timing of first occurrence of proteinuria compared with women who never had proteinuria in multivariable multinomial regression model (N = 11 651).**
(DOC)
