# Supplementary material for: Relationships of Risk Factors for Pre-Eclampsia with Patterns of Occurrence of Isolated Gestational Proteinuria during Normal Term Pregnancy
Source: PLoS One. 2011 Jul 18;6(7):e22115. doi: 10.1371/journal.pone.0022115 (PMC3138774; doi:10.1371/journal.pone.0022115)
Supplement: File S1 — Supplemental Methods: Description of the latent class analysis. (DOC) [file pone.0022115.s001.doc]

**S1 Supplemental Methods: Description of the latent class analysis**

Length of gestation was initially divided into 8 categories: ≤12 weeks, 13-16 weeks, 17-20 weeks, 21-24 weeks, 25-28 weeks, 29-32 weeks, 33-36 weeks and ≥37 weeks and the maximum amount of proteinuria which each woman had on any measurement in each gestational period was derived, or recorded as missing if the woman had no proteinuria measurements in that period. For the whole cohort of women (N=11,651) latent class models with 1 to 8 classes were fitted to 8 binary variables defining maximum proteinuria as “nil/trace” or “1+ or more” in each of the periods of gestational age. We aimed to select the model which minimised the Bayesian Information Criterion (BIC) and maximised the model entropy, whilst satisfying the assumption that variables were independent conditional on the latent class, which was measured by the overall chi-squared value for the bivariate residuals. However since few women had any proteinuria prior to 20 weeks gestation the bivariate residuals for maximum proteinuria in the first three periods of gestational age conditioning on class were large and the conditional independence assumption did not hold for any of the models fitted. To improve the model fit we combined the first three categories of gestational age to give 6 categories: ≤20 weeks, 21-24 weeks, 25-28 weeks, 29-32 weeks, 33-36 weeks and ≥37 weeks and 6 binary variables for maximum proteinuria in each of these categories. This improved the conditional independence assumption of the models. The model fit statistics of models with 1 to 8 classes using 6 periods of gestational age are shown in Table 1 below and the model with 2 latent classes was selected.

Further latent class analysis of women who ever had proteinuria of 1+ or more in pregnancy (N = 1122) was then performed to define different patterns of occurrence of proteinuria within these women. For this analysis maximum degree of proteinuria in each of the 6 periods of gestation was defined in three categories as “nil/trace”, “1+” or “2+ or more”. Models with 1 to 8 latent classes were fitted and Table 2 shows the model fit statistics for each of the models with 1 to 8 classes. We chose the model with five latent classes, since this had a similar BIC and entropy to the six class model (which had the lowest BIC), was the simplest model to satisfy the conditional independence assumption and had a higher percentage of women in the smallest class than the model with six classes. In the selected latent class models for the whole cohort and for only women who ever had proteinuria each woman was assigned a probability of belonging to each of the latent classes and random samples from the distributions of these class membership probabilities “pseudo-class draws”[1] were used to capture the uncertainty in class membership in subsequent logistic and multinomial regression models.

Table Model fit statistics for models with 1 to 8 classes for the whole cohort of women (N = 11 651)

| Number of Classes | Log-likelihood | BIC | Adjusted BIC | Entropy | Overall bivariate log-likelihood χ^2^ (df=15) | | Percentage of total in smallest class |
| --- | --- | --- | --- | --- | --- | --- | --- |
| 1 | -5934.60 | 11925.38 | 11906.31 | 1.00 | 223.7 | *P* < 0.001 | 100.00 |
| 2 | -5790.69 | 11703.09 | 11661.78 | 0.89 | 8.5 | *P* = 0.90 | 1.20 |
| 3 | -5781.34 | 11749.94 | 11686.38 | 0.87 | 4.0 | *P* = 0.998 | 0.05 |
| 4 | -5775.93 | 11804.66 | 11718.86 | 0.90 | 3.1 | *P* > 0.999 | 0.03 |
| 5 | -5771.17 | 11860.69 | 11752.64 | 0.94 | 0.7 | *P* > 0.999 | 0.03 |
| 6 | -5767.94 | 11919.77 | 11789.48 | 0.80 | 0.4 | *P* > 0.999 | 0.03 |
| 7 | -5766.26 | 11981.94 | 11829.40 | 0.81 | 0.3 | *P* > 0.999 | 0.02 |
| 8 | -5765.23 | 12045.42 | 11870.64 | 0.96 | 0.3 | *P* > 0.999 | 0.02 |

Table Model fit statistics for models with 1 to 8 classes for women who ever had proteinuria of 1+ or more (N = 1122)

| Number of Classes | Log-likelihood | BIC | Adjusted BIC | Entropy | Overall bivariate log-likelihood χ^2^ (df=60) | | Percentage of total in smallest class |
| --- | --- | --- | --- | --- | --- | --- | --- |
| 1 | -3281.79 | 6647.86 | 6609.74 | 1.00 | 317.8 | *P* < 0.001 | 100.00 |
| 2 | -3040.62 | 6256.81 | 6177.41 | 0.83 | 164.1 | *P* < 0.001 | 49.64 |
| 3 | -2935.94 | 6138.75 | 6018.05 | 0.97 | 113.7 | *P* < 0.001 | 26.56 |
| 4 | -2862.22 | 6082.61 | 5920.62 | 0.97 | 101.3 | *P* < 0.001 | 12.92 |
| 5 | -2804.09 | 6057.64 | 5854.36 | 0.95 | 61.5 | *P* = 0.42 | 9.00 |
| 6 | -2755.57 | 6051.90 | 5807.33 | 0.97 | 60.8 | *P* = 0.45 | 3.39 |
| 7 | -2724.39 | 6080.84 | 5794.97 | 0.98 | 17.3 | *P* > 0.999 | 0.62 |
| 8 | -2741.10 | 6206.29 | 5879.14 | 0.97 | 43.2 | *P* = 0.95 | 0.44 |

Note: Tables 1 and 2 are included as part of S1 as they are part of the supplemental methods and not referred to in the main text.

Reference List

1. Wang CP, Brown CH, Bandeen-Roche K (2005) Residual diagnostics for growth mixture models: Examining the impact of a preventive intervention on multiple trajectories of aggressive behavior. J Am Stat Assoc 100: 1054-1076.
