# Supplementary material for: Relationships of Risk Factors for Pre-Eclampsia with Patterns of Occurrence of Isolated Gestational Proteinuria during Normal Term Pregnancy
Source: PLoS One. 2011 Jul 18;6(7):e22115. doi: 10.1371/journal.pone.0022115 (PMC3138774; doi:10.1371/journal.pone.0022115)
Supplement: File S2 — Supplemental Methods: Development of the linear spline random effects models for blood pressure. (DOC) [file pone.0022115.s002.doc]

**S2 Supplemental Methods: Development of the linear spline random effects models for blood pressure**

The data were divided into 2 week intervals by gestational age, starting from 4 weeks gestation, and in cases where an individual had multiple blood pressure measurements within any 2 week interval, one measurement was chosen at random from this interval for inclusion in the sample for analysis. This was to prevent individuals with a high number of antenatal visits from having too great an influence on the models. After this process there remained a median of 10 blood pressure measurements per woman, with a range of 1 to 18.

Fractional polynomial curves, as described by Royston[1], were fitted to the data to describe the shape of the average pattern of blood pressure change with gestational age . Fractional polynomials are similar to conventional polynomials such as quadratic or cubic curves, but have a wider (infinite) range of possible powers of X and so provide more flexibility in shape. The models take the form Y = β_0_ + β_1_X^P1^ + β_2_X^P2^ + ...+ β_n_X^Pn^, where P1, ..., Pn are powers of time, X; we restricted to the powers: -2, -1, -0.5, 0, 0.5, 1, 2, 3 and considered fractional polynomials up to degree 2, meaning that up to two powers of time could be included in the models. This set of powers and degree of polynomial has been found to be sufficient to adequately describe most data.[1] Separate models were fitted with SBP and DBP as the outcome variables (Y) and for each model gestational age in weeks was used as the exposure variable (X). The models had two levels: antenatal visit and individual, since there were multiple antenatal visits per woman. An individual-level random effect was included and the powers of time were also allowed to vary at the individual level. The best-fitting model was selected as the model with the highest log-likehood.

We used the shape of the best-fitting fractional polynomial model for each of SBP and DBP to determine the approximate position and number of knot points in a linear spline random effects model. Models with 2 or 3 knots were considered and the final positioning and number of knots was selected as that which optimally fulfilled the criteria of a high model log-likelihood, a close fit to the fractional polynomial curve and good fit of the model predicted values to observed values over the whole course of pregnancy. The linear spline models had two levels, as above, and each contained an individual-level random effect and random slope parameters on each of the splines. The selected models for both SBP and DBP had 3 knots at 18, 30 and 36 weeks gestation. These main models were used to describe the average patterns of SBP and DBP change for the whole cohort of women. 90% of the predicted values lay within around 14 mmHg of the actual measurements for the SBP model and within around 10 mmHg of the actual measurements for the DBP model for each 2 week period of gestation. The chosen spline models had the following equations:

$$SBP_{ij}=112.20+(-0.138+u_{1j})s_{1ij}+(0.154+u_{2j})s_{2ij}+(0.277+u_{3j})s_{3ij}+(1.121+u_{4j})s_{4ij}+u_{0j}+\varepsilon_{0ij}$$

$$DBP_{ij}=66.03+(-0.186+u_{1j})s_{1ij}+(0.107+u_{2j})s_{2ij}+(0.458+u_{3j})s_{3ij}+(1.289+u_{4j})s_{4ij}+u_{0j}+\varepsilon_{0ij}$$

for the ith measurement on the jth individual where *u*_0j_, .., *u*_4j_ are the individual level residuals (random effects); *ε*_0ij_ is the measurement occasion level residual; s_1_ is the first spline for gestational age, up to 18 weeks gestation; s_2_ is the second spline, from 18-30 weeks; s_3_ is the third spline, from 30-36 weeks; and s_4_ is the fourth spline, from 36 weeks onwards. The intercept was at 8 weeks gestation.

Reference List

1. Royston P, Altman DG (1994) Regression Using Fractional Polynomials of Continuous Covariates - Parsimonious Parametric Modeling. Applied Statistics-Journal of the Royal Statistical Society Series C 43: 429-467.
