# Supplementary material for: Relationships of Risk Factors for Pre-Eclampsia with Patterns of Occurrence of Isolated Gestational Proteinuria during Normal Term Pregnancy
Source: PLoS One. 2011 Jul 18;6(7):e22115. doi: 10.1371/journal.pone.0022115 (PMC3138774; doi:10.1371/journal.pone.0022115)
Supplement: Table S3 — Probabilities of class membership (analysis of women who ever had proteinuria) and membership of the subgroup of women who never had proteinuria in extreme categories of the maternal risk factors from the multivariable multinomial regression model (N = 8915). (DOC) [file pone.0022115.s003.doc]

Table S3 Probabilities of class membership (analysis of women who ever had proteinuria) and membership of the subgroup of women who never had proteinuria in extreme categories of the maternal risk factors from the multivariable multinomial regression model (N = 8915)a

| **Maternal Characteristic**  (Extreme categories) | **Women with no proteinuria** (91.1%) | | **Class 1** (0.84%)  Onset ≤ 20 weeks | | **Class 2** (0.87%)  Onset 21-28 weeks | | **Class 3** (1.08%)  Onset 29-32 weeks | | | **Class 4** (1.91%)  Onset 33-36 weeks | | **Class 5** (4.23%)  Onset 37+ weeks | |
| --- | --- | --- | --- | --- | --- | --- | --- | --- | --- | --- | --- | --- | --- |
|  | Probability  of belonging to subgroup | % of group | Probability of belonging to class | % of class | Probability of belonging to class | % of class | Probability  of belonging to class | | % of class | Probability  of belonging to class | % of class | Probability  of belonging to class | % of class |
| **Pre-pregnancy BMI (kg/m^2^)**  Underweight  Obese | 0.945  0.858 | 5.00  4.73 | 0.006  0.008 | 6.27  7.87 | 0.002  0.010 | 2.07  8.11 | 0.002  0.008 | 2.06  8.14 | | 0.017  0.023 | 6.92  10.23 | 0.027  0.093 | 2.36  8.11 |
| **Age (yrs)**  <20  35+ | 0.879  0.914 | 3.30  10.56 | 0.032  0.004 | 10.60  7.91 | 0.013  0.004 | 7.01  5.60 | 0.007  0.003 | 4.09  6.79 | | 0.008  0.010 | 2.79  9.14 | 0.061  0.065 | 3.96  11.04 |
| **Parity**  Nulliparous  Multiparous | 0.919  0.930 | 44.41  55.59 | 0.005  0.009 | 39.29  60.71 | 0.006  0.007 | 42.81  57.19 | 0.006  0.009 | 36.54  63.46 | | 0.011  0.012 | 41.15  58.85 | 0.053  0.033 | 54.92  45.08 |
| **Smoking during pregnancy**  Never  Throughout | 0.919  0.928 | 69.07  17.77 | 0.005  0.004 | 73.11  18.74 | 0.006  0.009 | 57.83  25.60 | 0.006  0.007 | 58.51  20.48 | | 0.011  0.011 | 64.45  18.19 | 0.053  0.041 | 73.28  15.24 |
| **Highest qualification**  CSE/vocational  Degree | 0.908  0.933 | 27.08  14.19 | 0.005  0.006 | 29.72  10.85 | 0.007  0.007 | 37.34  12.99 | 0.009  0.005 | 36.01  8.27 | | 0.015  0.008 | 35.62  8.39 | 0.056  0.041 | 28.26  11.28 |
| **Pregnancy type**  Male singleton  Twin | 0.919  0.800 | 50.62  0.65 | 0.005  <0.001 | 50.05  0.05 | 0.006  0.026 | 49.30  2.91 | 0.006  0.026 | 48.22  3.09 | | 0.011  0.083 | 41.39  4.38 | 0.053  0.064 | 51.02  0.87 |

^a^ Probabilities shown are for women in the reference category for each of the other variables: normal weight, aged 25-29 years, nulliparous, never smoked, O level qualification and male singleton pregnancy. The “% of group/class” is the percentage of women in each class who have the specified characteristic.
