# Supplementary material for: Relationships of Risk Factors for Pre-Eclampsia with Patterns of Occurrence of Isolated Gestational Proteinuria during Normal Term Pregnancy
Source: PLoS One. 2011 Jul 18;6(7):e22115. doi: 10.1371/journal.pone.0022115 (PMC3138774; doi:10.1371/journal.pone.0022115)
Supplement: Table S4 — Odds ratios for membership of subgroups defined by timing of first occurrence of proteinuria compared with women who never had proteinuria in multivariable multinomial regression model (N = 11 651). (DOC) [file pone.0022115.s004.doc]

Table S4 Odds ratios for membership of subgroups defined by timing of first occurrence of proteinuria compared with women who never had proteinuria in multivariable multinomial regression model (N = 11,651)^ab^

| **Maternal Characteristic** | **Subgroup 1**^b^ (1.07%)  “Onset ≤ 20 weeks” | | **Subgroup 2**^b^ (1.06%)  “Onset 21-28 weeks” | | **Subgroup 3**^b^ (1.12%)  “Onset 29-32 weeks” | | **Subgroup 4**^b^ (2.15%)  “Onset 33-36 weeks” | | **Subgroup 5**^b^ (4.23%)  “Onset 37+ weeks” | |
| --- | --- | --- | --- | --- | --- | --- | --- | --- | --- | --- |
|  | Odds ratio | 95% CI | Odds ratio | 95% CI | Odds ratio | 95% CI | Odds ratio | 95% CI | Odds ratio | 95% CI |
| **Pre-pregnancy BMI (kg/m^2^)**  Underweight  Normal  Overweight  Obese | 1.20  1  1.18  2.21 | (0.57, 2.51)  -  (0.69, 1.99)  (1.16, 4.20) | 0.64  1  1.28  1.51 | (0.17, 2.46)  -  (0.77, 2.12)  (0.68, 3.33) | 0.47  1  1.14  1.44 | (0.14, 1.63)  -  (0.68, 1.90)  (0.69, 3.00) | 1.42  1  1.40  1.76 | (0.81, 2.47)  -  (0.96, 2.03)  (1.02, 3.04) | 0.68  1  1.31  1.72 | (0.40, 1.17)  -  (1.01, 1.70)  (1.15, 2.56) |
| ***P* for all classes** = 0.026 |  | |  | |  | |  | |  | |
| **Age (yrs)**  <20  20-24  25-29  30-34  35+ | 3.89  2.41  1  1.02  0.83 | (1.96, 7.69)  (1.50, 3.85)  -  (0.61, 1.71)  (0.38, 1.82) | 1.88  1.33  1  1.19  0.71 | (0.92, 3.84)  (0.81, 2.16)  -  (0.74, 1.90)  (0.31, 1.60) | 1.82  1.54  1  0.93  0.56 | (0.84, 3.93)  (0.97, 2.44)  -  (0.59, 1.48)  (0.25, 1.26) | 1.25  1.33  1  0.91  0.84 | (0.70, 2.21)  (0.95, 1.85)  -  (0.65, 1.28)  (0.50, 1.41) | 1.10  1.10  1  1.02  1.25 | (0.71, 1.71)  (0.85, 1.42)  -  (0.80, 1.29)  (0.90, 1.73) |
| ***P* for all classes** = 0.002 |  | |  | |  | |  | |  | |
| **Parity**  Nulliparous  Multiparous | 1  1.25 | -  (0.83, 1.87) | 1  0.91 | -  (0.62, 1.34) | 1  1.60 | -  (1.06, 2.41) | 1  0.98 | -  (0.74, 1.30) | 1  0.63 | -  (0.52, 0.77) |
| ***P* for all classes** < 0.001 |  | |  | |  | |  | |  | |
| **Smoking during pregnancy**  Never  Pre-pregnancy/1^st^ trimester  Throughout | 1  0.73  1.01 | -  (0.40, 1.32)  (0.64, 1.59) | 1  1.29  1.59 | -  (0.76, 2.19)  (1.02, 2.47) | 1  1.50  1.23 | -  (0.91, 2.49)  (0.79, 1.93) | 1  1.38  1.27 | -  (0.95, 2.00)  (0.91, 1.77) | 1  0.76  0.88 | -  (0.56, 1.02)  (0.69, 1.14) |
| ***P* for all classes** = 0.107 |  | |  | |  | |  | |  | |
| **Highest qualification**  CSE/vocational  O level  A level  Degree | 1.04  1  1.24  0.98 | (0.65, 1.66)  -  (0.73, 2.09)  (0.47, 2.05) | 1.21  1  0.85  1.11 | (0.77, 1.88)  -  (0.46, 1.56)  (0.55, 2.22) | 1.38  1  1.56  0.76 | (0.86, 2.24)  -  (0.92, 2.62)  (0.31, 1.84) | 1.17  1  0.98  0.82 | (0.84, 1.63)  -  (0.66, 1.45)  (0.48, 1.41) | 0.96  1  0.95  0.75 | (0.76, 1.22)  -  (0.74, 1.23)  (0.54, 1.06) |
| ***P* for all classes** = 0.704 |  | |  | |  | |  | |  | |
| **Pregnancy type**  Male singleton  Female singleton  Twin | 1  0.96  -^c^ | -  (0.67, 1.37)  -^c^ | 1  0.89  4.84 | -  (0.62, 1.28)  (1.70, 13.73) | 1  1.17  6.73 | -  (0.82, 1.67)  (2.60, 17.43) | 1  1.18  5.68 | -  (0.91, 1.52)  (2.66, 12.15) | 1  1.10  1.31 | -  (0.92, 1.32)  (0.47, 3.62) |
| ***P* for all classes** < 0.001 |  | |  | |  | |  | |  | |

^a^ Missing covariate data was imputed using multivariate multiple imputation. Chained equations were used to produce 20 full datasets and model coefficients were averaged over the datasets using Rubin’s rules[1] to produce standard errors. The outcome, covariates and predictors of missingness were included in prediction models to impute data.

^b^ Subgroups were defined according to the gestational age when the woman’s first proteinuria measurement of 1+ or more occurred, in categories of: ≤20 weeks, 21-28 weeks, 29-32 weeks, 33-36 weeks and 37+ weeks gestation. The comparison group is women who had no proteinuria measurements of 1+ or more in pregnancy (90.37%)

^c^ Odds ratio could not be estimated as there were too few women with this characteristic in this subgroup

Reference List

1. Royston P (2004) Multiple imputation of missing values. Stata Journal 4: 227-241.
